# Impact of climate change on the geographical distribution and niche dynamics of *Gastrodia elata*

**DOI:** 10.7717/peerj.15741

**Published:** 2023-07-24

**Authors:** Juan Hu, Ying Feng, Haotian Zhong, Wei Liu, Xufang Tian, Yehong Wang, Tao Tan, Zhigang Hu, Yifei Liu

**Affiliations:** 1 College of Pharmacy, Hubei University of Chinese Medicine, Wuhan, China; 2 Wufeng Tujia Autonomous County Agricultural Science and Technology Demonstration Center, Yichang, China; 3 Wufeng Tujia Autonomous County Herbal Medicine Development Center, Yichang, China

**Keywords:** *Gastrodia elata*, Climate change, Highly suitable area, Ecological niche

## Abstract

**Background:**

*Gastrodia elata* is widely used in China as a valuable herbal medicine. Owing to its high medicinal and nutrient value, wild resources of *G. elata* have been overexploited and its native areas have been severely damaged. Understanding the impacts of climate change on the distribution of this endangered species is important for the conservation and sustainable use of *G. elata*.

**Methods:**

We used the optimized maximum entropy model to simulate the potential distribution of *G. elata* under contemporary and future time periods (1970–2000, 2050s, 2070s, and 2090s) and different climate change scenarios (SSP1-2.6, SSP2-4.5, SSP3-7.0, and SSP5-8.5). Under these conditions, we investigated the key environmental factors influencing the distribution of *G. elata* as well as the spatial and temporal characteristics of its niche dynamics.

**Results:**

With high Maxent model accuracy (AUCmean = 0.947 ± 0.012, and the Kappa value is 0.817), our analysis revealed that annual precipitation, altitude, and mean temperature of driest quarter are the most important environmental factors influencing the distribution of *G. elata*. Under current bioclimatic conditions, the potentially suitable area for *G. elata* in China is 71.98 × 10^4^ km^2^, while the highly suitable region for *G. elata* growth is 7.28 × 10^4^ km^2^. Our models for three future periods under four climate change scenarios indicate that *G. elata* can maintain stable distributions in southern Shaanxi, southwestern Hubei, and around the Sichuan basin, as these areas are highly suitable for its growth. However, the center of the highly suitable areas of *G. elata* shift depending on different climatic scenarios. The values of niche overlap for *G. elata* show a decreasing trend over the forecasted periods, of which the niche overlap under the SSP3-7.0 scenario shows the greatest decrease.

**Discussions:**

Under the condition of global climate change in the future, our study provides basic reference data for the conservation and sustainable utilization of the valuable and endangered medicinal plant* G. elata*. It is important to carefully choose the protection area of *G. elata* wild resources according the suitable area conditions modeled. Moreover, these findings will be valuable for providing insights into the breeding and artificial cultivation of this plant, including the selection of suitable areas for planting.

## Introduction

Climate change is the most important factor affecting both the contemporary and future distributions of plant species (Zhao et al., 2021a; Zhao et al., 2021b). Greenhouse gases emitted by human activities have not only raised air temperatures, but also significantly altered the functioning of ecosystems. According to the Sixth Assessment Report of the Intergovernmental Panel on Climate Change (IPCC), surface temperatures have risen at an unprecedented rate since the 1970s (Zhou, 2021). For example, between 1909 and 2011, the average temperature over land areas in China rose by 0.9−1.5 °C, and is predicted to further rise by another 1.3 to 5.0 °C by the end of the 21st century (Liu et al., 2021; Zhao et al., 2021a; Zhao et al., 2021b). In response to climate change, the distribution patterns of many species are changing (García-Valdés et al., 2013; Pecl et al., 2017; Scheffers et al., 2016). It is imperative to predict the potential effects of climate change on the potential distributions of ecologically and economically important plant species so as to facilitate their conservation and sustainable use.

Ecological niche models (ENMs) have been widely used to assess and predict the impacts of climate change on species’ distributions (Warren, Glor & Turelli, 2010). Using statistical or theoretical methods, ENMs combine data on species’ distributions with environmental variables to obtain response surfaces with the aim of describing, understanding and predicting species distributions (Sillero et al., 2021). Ecological models that are currently commonly used for predicting species distributions include the genetic algorithm for rule-set prediction (Garp), the maximum entropy model (Maxent), the bioclimatic model (Bioclim), and the geographic information system for global medicinal plant (GMPGIS) (Austin, 2002; Beaumont, Hughes & Poulsen, 2005; Booth, 2018; Feng et al., 2020). Many studies across a range of contexts—such as biodiversity conservation, pest control, and alien species invasions—have shown that the Maxent model affords high power and stability for predicting the current and future distribution of species (Hampe & Petit, 2005; He et al., 2022; Wu et al., 2021; Xu et al., 2020).

*Gastrodia elata* Blume (Orchidaceae) is a perennial and mycoheterotrophic plant species (Xu et al., 2021). *Gastrodia elata* is sensitive to high and low temperature extremes, and as such is typically found growing in environments that are cool during the summer and warm during the winter. The optimum temperatures for the growth of *G. elata* range from 15 to 25 °C (Yuan et al., 2020). In China, *G. elata* has a relatively extensive native distribution that stretches across several provinces; these include Yunnan, Shaanxi, Guizhou, Sichuan, Hubei, Jilin, and Anhui. *G. elata* grows in montane forests that range between 700 to 3,200 m in altitude (Wang et al., 2019a; Wang et al., 2019b). As a traditional form of herbal medicine, *G. elata* has long been a subject of cultural and scientific interest. In traditional Chinese medicine (TCM), the dried rhizomes of *G. elata* have been used as a treatment for headaches, dizziness, convulsions, epilepsy, rheumatism, and neurasthenia (Ahn et al., 2007; Li et al., 2020; Wang et al., 2021). Modern pharmacological studies have also shown that *G. elata* can be used as a treatment for sleep disturbances and memory loss (Heese, 2020; Huang et al., 2021). With increasing demands for *G. elata* in the commercial market, native populations of *G. elata* have been seriously impacted by overexploitation (Chen et al., 2014).

In this study, we simulated the potential distribution of *G. elata* using Maxent models. Specifically, we analyze and predict the distributions of potentially suitable regions for *G. elata* under multiple climate change scenarios (SSP1−2.6, SSP2−4.5, SSP3−7.0, and SSP5−8.5) in contemporary and future time periods (1970–2000, 2050s, 2070s, and 2090s) (Zhao et al., 2021a; Zhao et al., 2021b). We identified environmental factors determining the distribution of *G. elata* populations, and further predicted the highly suitable areas of *G. elata* under climate change. Finally, we identified areas where conservation of wild *G. elata* resources should be prioritized. Overall, we aim to provide empirical knowledge on the ecology and distribution of *G. elata* that can guide the conservation and sustainable cultivation of this species in China.

## Methods

### Data on species distribution

Data on the distribution of *G. elata* in China was obtained from a preliminary field investigation and a literature review. The latter included distribution records from the Chinese Virtual Herbarium (CVH), the Global Biodiversity Information Facility (GBIF), the Specimen Resource Sharing Platform for Education (SRSPE), and the Plant Photo Bank of China (PPBC). To minimize errors caused by sampling, we deleted duplicate records and spatially filtered the remaining data points (Varela et al., 2014; Warren, Glor & Turelli, 2010). Consequently, a maximum of one occurrence record was assigned to each 3 ×3  km grid cell. A final dataset of 277 occurrence records of *G. elata* were collected (Table S1).

### Filtering of environmental factors

In this study, 19 bioclimatic factors and elevation variables were obtained from WorldClim, covering current (1970–2000) and three future time periods (the 2050s, 2070s, and 2090s). The bioclimatic factors of future climate included four different climate change scenarios (SSPs; SSP1−2.6, SSP2−4.5, SSP3−7.0, and SSP5−8.5) (Riahi et al., 2017). The general circulation model by Beijing Climate Center Climate System Model (BCC-CSM2-MR) was adopted for bioclimatic data corresponding to future time periods (O’Neill et al., 2017). Consequently, our analysis included a total of 13 sets of environmental data (*i.e.,* one set describing the current environment and 12 sets describing future environments). The environmental data had a spatial resolution of 2.5 arc minutes and was converted to an ASCII file format by using ArcGIS10.4.1.

The selected environmental variables can strongly influence the accuracy of a species distribution model (Wang et al., 2019a; Wang et al., 2019b). As strong correlations among multiple environmental variables would cause over-fitting of model predictions and undermine the accuracy of the model results, we excluded strongly correlated environmental variables based on the Pearson correlation coefficients (*r*). We evaluated the importance of environmental variables by the Jackknife method and considered two environmental variables to exhibit high collinearity if —*r —*>0.8 (Liu et al., 2021). Accordingly, we used a final set of 11 environmental variables to build the species distribution models (Table 1).

**Table 1:** Environmental variables used for modeling and their permutation rates.

**Environmental variable**	**Code**	**Percent contribution**
Annual precipitation	bio12	49.3
Altitude	alt	24.6
Mean Temperature of Driest Quarter	bio9	10.1
Isothermality	bio3	3.7
Mean diurnal air temperature range	bio2	3.1
Mean temperature of coldest quarter	bio11	1
Air temperature seasonality	bio4	0.2
Max temperature of warmest month	bio5	0.2
Min temperature of coldest month	bio6	0.2
Precipitation seasonality	bio15	0.2
Precipitation of coldest quarter	bio19	0.2

### Model establishment and optimization

We used Maxent v3.4.1 software to analyze and forecast the distribution of suitable areas for *G. elata.* We sought to establish a distribution of *G. elata* that would closely follow a normal distribution. Hence, the model was trained with 75% of the distribution data for *G. elata* and tested with the remaining 25% of the distribution data to verify its accuracy (Phillips et al., 2017). To further optimize the prediction quality of the model, we set a maximum of 1,000 background points and set all other settings to default.

We used the *kuenm* package in R v4.1.3 (R Core Team, 2022) to further optimize the feature class (FC) and regularization multiplier (RM) of the model (Cobos et al., 2019). The feature class contains five element types, including Linear (L), Quadratic (Q), Hinge (H), Product (P), and Threshold (T) element types. These element types were arranged and combined to form 31 FC combinations. We set values of RM to range between 0.1-2 at an interval of 0.1. This allowed us to produce 620 parameter combinations of FC and RM. Finally, Maxent modeling is performed when the optimal parameter combination is *δ*AICc = 0 (Cobos et al., 2019; Korbel, 2021; Zhuo et al., 2020).

### Validating model reliability and classification of suitable areas

We evaluated the accuracy of a model’s prediction based on the area under the receiver operating characteristic (ROC) curve (AUC) (Warren & Seifert, 2011). We also evaluated the relative significance of each parameter using Jackknife test. The values of AUC range from 0 to 1, which are positively correlated with the accuracy of a model’s predictions. The AUC value above 0.9 indicates that a model very accurately predicts the target outcome (Liu et al., 2019). At the same time, we use the *NicheToolBox* package to calculate Kappa value (Luis et al., 2020). Kappa statistic is a consistency test method widely used in model evaluation. When the Kappa value is great than 0.6, the consistency is significant, and the larger the value, the higher the prediction accuracy (Monserud & Leemans, 1992).

The Maxent model predicted the potential distribution areas of *G. elata* in China. We further classified these areas based on their ecological suitability for the species. The species presence probability values were classified by manual classification methods through the reclassification function of ArcGIS10.4.1. Potentially suitable areas were classified into four categories: highly suitable areas (0.7-1), moderately suitable areas (0.5−0.7), lowly suitable areas (0.3−0.5), and unsuitable areas (0−0.3).

### Spatial variation of highly suitable areas and shifts in distribution centers

We used the SDM toolbox in ArcGIS10.4.1 to further calculate the potential high suitable areas and suitable area distribution center of *G. elata* in the future scenarios (Brown & Anderson, 2014). We reclassified spatial units that had a species distribution probability value ≥ 0.7 as the most suitable areas for *G. elata*, and <0.7 as the unsuitable areas. This allowed us to construct an existence/nonexistence (0, 1) matrix for *G. elata* (Zhao et al., 2021a; Zhao et al., 2021b). We investigated whether spatial variations in the distribution of *G. elata* showed one of three patterns: expansion, contraction, and no change.

Based on the statistical parameters of the distribution of high suitability areas, we narrowed the distribution range of *G. elata* down to a single central point, termed the center of mass. We determined and compared the locations and directions of changes in the center of mass for highly suitable areas in different time periods (the present, the 2050s, the 2070s, and the 2090s) using the SDM toolbox in ArcGIS10.4.1 software. Finally, we calculated the spatial distances by which highly suitable areas for *G. elata* shifted based on the latitudinal and longitudinal coordinates of the center of mass.

### Calculating climatic niche characteristics

To characterize and compare current and future changes in the climatic niche of *G. elata*, we used a method based on principal component analysis (PCA) from the *ecospat* package in R v4.1.3 (Broennimann et al., 2012) to transform the environmental variables into a dimensional space defined by the first two components PC1 and PC2. This two-dimensional space was established on a 100 × 100 cell grid based on the minimum and maximum PCA values of environmental data. A kernel density function was further used to estimate the smoothed density of species occurrences in each cell within the grid (Di Cola et al., 2017). We then used Schoener’s *D* metric to calculate values of niche overlap, which ranged between 0 (no similarity) and 1 (complete similarity) (Hamid et al., 2018; Quiroga, Premoli & Fernandez, 2018). Using the values of niche overlap, we then compared the characteristics of ecological niches of *G. elata* under four climate change scenarios for the present and three future time periods.

## Results

### Model accuracy

By including a total of 277 distribution locations and 11 environment variables in our Maxent niche model, we were able to predict the distribution of *G. elata* in China (Table S1). We created 620 candidate models by including all 31 possible combinations of five FCs and 20 values of RM. Following model optimization, the best FC combination was PH with an RM value of 1.6. The results showed that the optimized parameters reduced the fit and complexity of the model. A high AUC value indicates that a model is performing better within the predicted distribution (Warren & Seifert, 2011). The mean AUC value of 10 replicate runs for our model was 0.947 with a standard deviation of 0.012, and the Kappa value was 0.817 (Fig. S1). This indicated that our model was accurate in predicting the distribution of potentially suitable areas for *G. elata* in China.

### Environmental factors influencing the distribution of *G. elata*

Several environmental variables had similar properties and high statistical correlations. To avoid data redundancy, we performed a correlation analysis of the environmental variables and a selection of dominant climatic variables from the results obtained by Jackknife (Tang et al., 2021). We then selected 11 environmental variables to include in the prediction model (Table 1). The three environmental variables that contributed most to model predictions were annual precipitation (49.3%), altitude (24.6%), and mean temperature of driest quarter (10.1%); collectively, these variables accounted for 84.2% of model predictions (Table 1). The remaining variables—which included isothermality, mean diurnal air temperature range, mean temperature of coldest quarter, air temperature seasonality, max temperature of warmest month, min temperature of the coldest month, precipitation seasonality, and precipitation of coldest quarter—showed low contributions to model predictions, indicating their limited influence on the distribution of suitable areas for *G. elata*. We also evaluated environmental factors affecting the potential distribution of *G. elata* using the Jackknife method, which revealed that the most important environmental factors were the min temperature of the coldest month, the mean temperature of the coldest quarter, and annual precipitation (Fig. S2). Based on the response curves of main environmental variables, our model identified the suitable area conditions for *G. elata* at present, which are as follows: annual precipitation ranging from 741.81 to 1,266.47 mm, a mean temperature of the driest quarter ranging from −0.49 to 6.23 °C, a mean temperature of coldest quarter ranging from −0.94 to 5.73 °C and an altitude ranging from 767 to 2,394 m (Fig. S3).

### The potential distribution of *G. elata* at present

**Figure 1:**
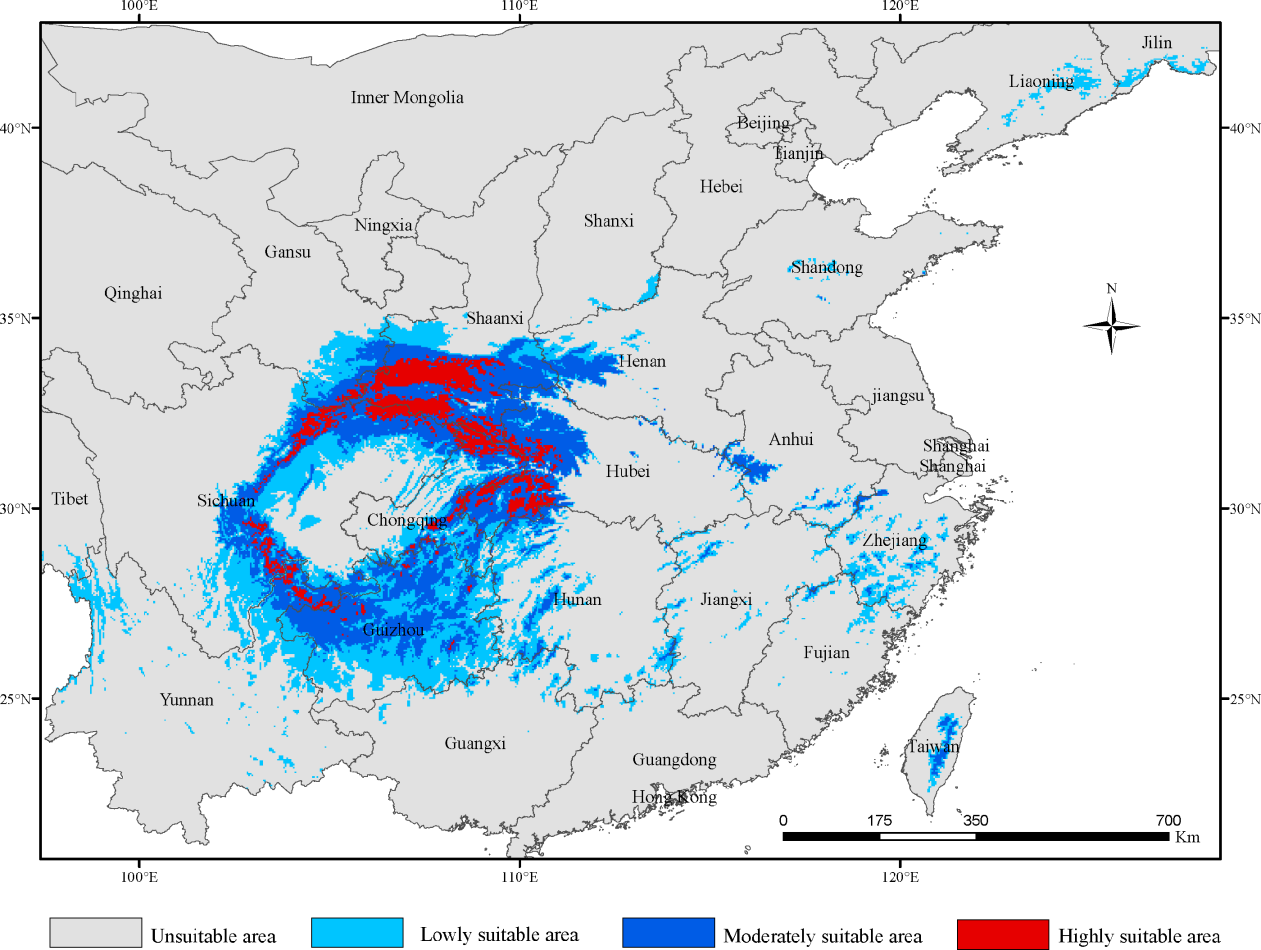
The distribution of potentially suitable areas for *Gastrodia elata* under current climatic scenarios.

The total area of potentially suitable areas of *G. elata* in China under current bioclimatic conditions was 71.98 × 10^4^ km^2^, of which the highly suitable areas was 7.28 × 10^4^ km^2^, the moderately suitable areas was 27.25 ×10^4^ km^2^ and the lowly suitable areas was 37.45 × 10^4^ km^2^ (Table 2). The potential suitable areas mainly included areas in the provinces of Gansu, Sichuan, Chongqing, Hubei, Shaanxi, Yunnan, Guizhou, Hunan, Henan and Anhui (Fig. 1). In addition, we found more patchily distributed areas suitable for *G. elata* growth located in Jilin, Liaoning, Jiangxi, Shandong, Shanxi, Guangxi, Tibet and Taiwan. The highly suitable areas for *G. elata* in China located in Hubei, Shaanxi, Chongqing, and Sichuan, as well as a few locations in southeastern Gansu, northeastern Yunnan, and southwestern Guizhou. By comparison, the moderately suitable areas were mainly distributed in Guizhou and northwestern Hubei, and the lowly suitable areas were mainly distributed around the areas of moderately suitable areas. Overall, the suitable areas for *G. elata* were primarily distributed across the second step of China’s terrain, with high suitability areas being primarily located in the mountains around the Sichuan Basin, such as Qinling Mountain, the Ta-pa Mountains and the Hengduan Mountains.

**Table 2:** Areas of potential distribution of *Gastrodia elata* under different climate scenarios.

**Decades**		**Predicted Area (**×**10**^**4**^**km**^**2**^**) and % of the Corresponding Current Area**			
		Low Suitable Region	Moderate Suitable Region	High Suitable Region	Total Suitable Region
current		37.45	27.25	7.28	71.98
SSP1-2.6	2050s	40.38 (107.82%)	27.84 (102.16%)	6.56 (90.10%)	74.78 (103.88%)
	2070s	39.11 (104.43%)	27.59 (101.24%)	4.57 (62.77%)	71.27 (99.01%)
	2090s	39.55 (105.61%)	30.34 (111.33%)	5.33 (73.21%)	75.22 (104.50%)
SSP2-4.5	2050s	39.79 (106.24%)	31.82 (116.77%)	5.02 (68.95%)	76.63 (106.46%)
	2070s	38.19 (101.97%)	24.25 (88.99%)	6.39 (87.77%)	68.83 (95.62%)
	2090s	38.04 (101.57%)	30.19 (110.78%)	5.72 (78.57%)	73.95 (102.73%)
SSP3-7.0	2050s	40.90 (109.21%)	29.42 (107.96%)	5.85 (80.35%)	76.17 (105.82%)
	2070s	41.20 (110.01%)	26.75 (98.16%)	4.89 (67.17%)	72.84 (101.19%)
	2090s	39.35 (105.07%)	28.39 (104.18%)	6.37 (87.50%)	74.11 (102.95%)
SSP5-8.5	2050s	39.67 (105.93%)	29.68 (108.91%)	5.83 (80.08%)	75.18 (104.44%)
	2070s	37.61 (100.42%)	26.29 (96.47%)	5.86 (80.49%)	69.76 (96.91%)
	2090s	41.17 (109.93%)	28.30 (103.85%)	6.38 (87.63%)	75.85 (105.37%)

### Changes in the distribution of potential and highly suitable areas for *G. elata* under future climatic conditions

**Figure 2:**
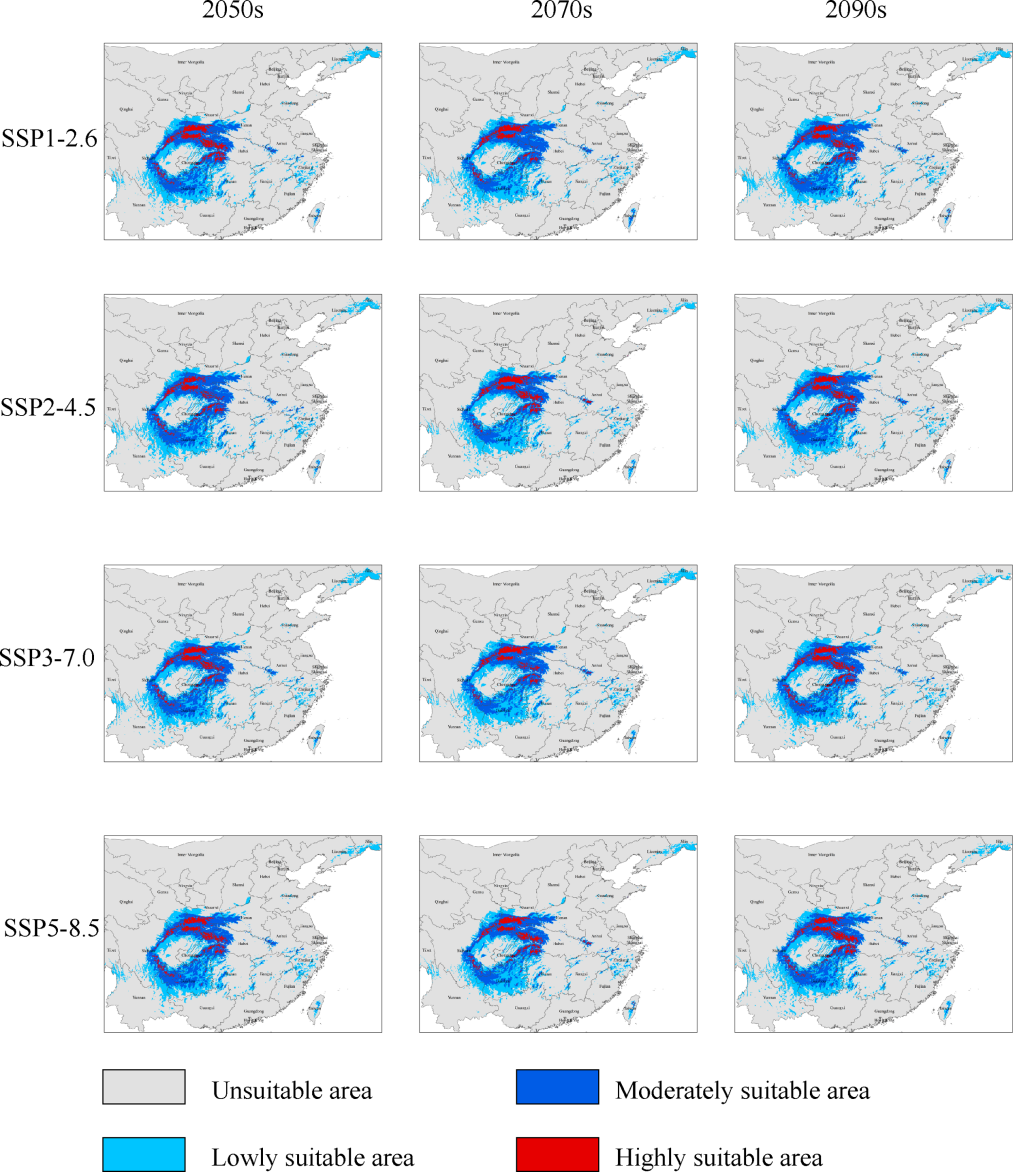
The distributions of potentially suitable areas for *Gastrodia elata* under different climate change scenarios.

We modelled the distributions of potentially suitable areas for *G. elata* in three future time periods under four climate change scenarios, and used the SDM toolbox to extract the future distributions of highly suitable areas for comparisons with their current distribution. We found that the distribution of potentially suitable areas for *G. elata* under the different scenarios revealed a similar trend, in which the total suitable area is expected to expand in the 2050s, contract to its lowest point in the 2070s, before once again expanding in the 2090s (Fig. 2, Table 2). In comparison with the current period, the future distribution of highly suitable areas all shows a decreasing trend. These results indicate that the contraction of the range of *G. elata* in the future will substantially exceed any expansions of its range (Table S2).

**Figure 3:**
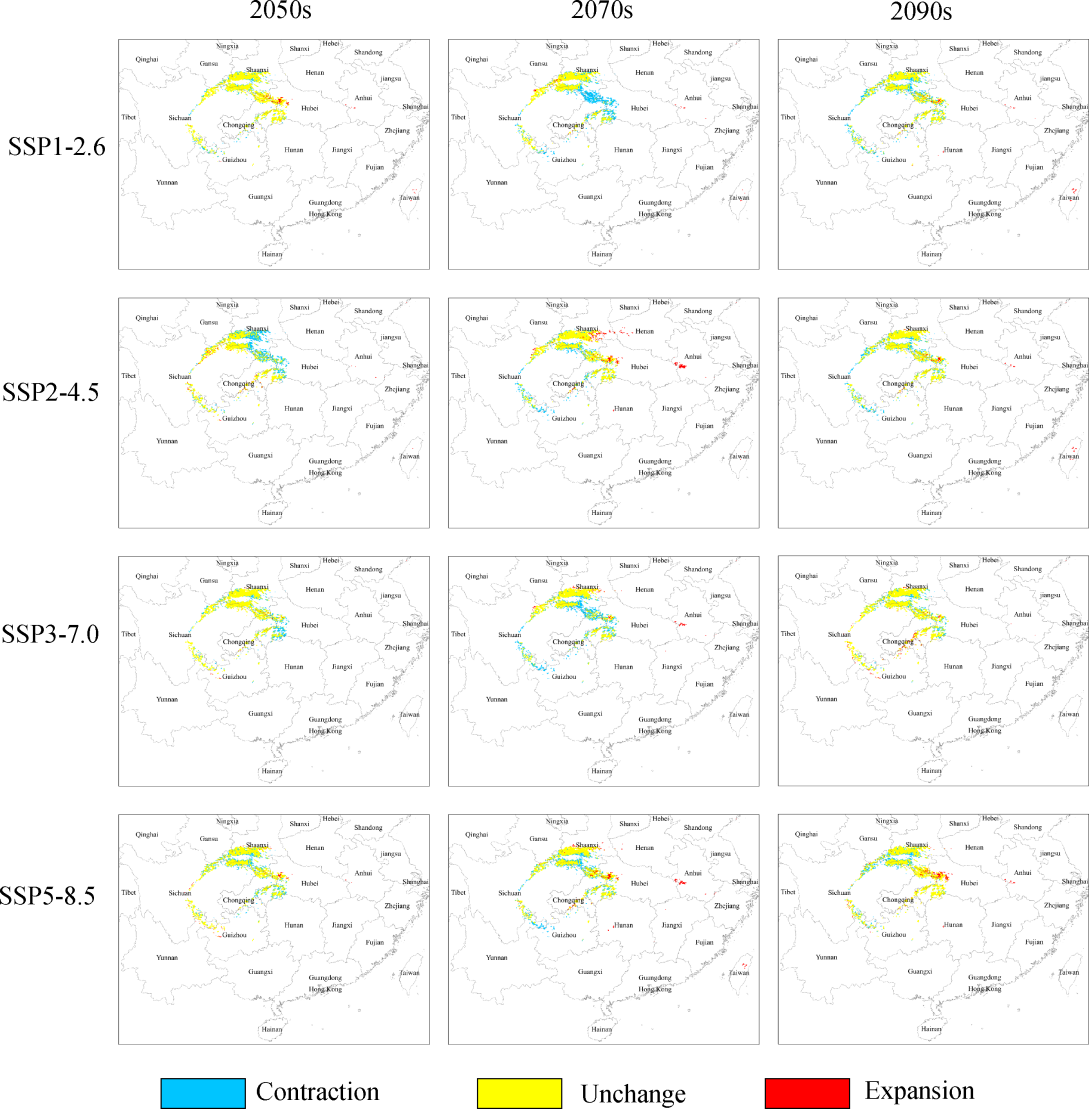
Changes in the distributions of highly suitable areas for *Gastrodia elata* in future periods under different climate change scenarios.

Under the SSP1−2.6 scenario, the total distribution of potentially suitable areas for *G. elata* in the three future time periods is expected to constitute 74.78 × 10^4^ km^2^ (in the 2050s), 71.27 × 10^4^ km^2^ (in the 2070s), 75.22 × 10^4^ km^2^ (in the 2090s), which accounted for 103.88%, 99.01%, and 104.50% of the current corresponding values, respectively (Table 2). The distribution of the total suitable area is expected to expand in the 2050s, with the most significant expansions occurring within low suitable areas, which will mainly be located within the Jilin and Liaoning provinces. The distributions of highly suitable areas are expected to contract most extensively during the 2070s, with this range contraction mainly occurring at the intersection of Hubei, Shaanxi, and Chongqing (Fig. 3). In comparison with their current distributions, the future distributions of both lowly and moderately suitable areas are expected to expand in the 2090s.

Under the SSP2−4.5 scenario, the total suitable areas (76.63 × 10^4^ km^2^) for *G. elata* are expected to expand most during the 2050s in comparison with the two other future time periods (Table 2). These areas are expected to contract to its lowest point during the 2070s. It will constitute only 68.83 × 10^4^ km^2^ which account for 95.62% of the currently total suitable areas. Between the 2050s and the 2070s, several areas in Guizhou province are expected to deteriorate from moderately suitable areas to lowly suitable areas. The current distribution of highly suitable areas across southeastern Shaanxi, Chongqing, and Hubei is expected to contract significantly and transition to moderately suitable areas by the 2050s. Subsequently, the distribution of highly suitable areas is expected to expand in eastern Shaanxi and western Hubei, while also emerging in the Dabie Mountains of Anhui by the 2070s (Fig. 3).

Under the SSP3−7.0 climate change scenario, the total distribution of potentially suitable areas for *G. elata* shows an overall trend of expansion. In the 2050s, the contraction of highly suitable areas will mainly occur in the west of Hubei, while the expansion of lowly and moderately suitable areas will occur in the northeast of Yunnan. In the 2070s, the distribution of highly suitable areas is expected to contract significantly to an area of 4.89 × 10^4^ km^2^ which account for 67.17% of the currently highly suitable areas. Such range contraction of highly suitable areas will primarily occur at the junction of Shaanxi and Chongqing, while the expansion of highly suitable areas will occur in the middle of Guizhou (Fig. 3).

Under the SSP5−8.5 climate change scenario, the total distribution of potentially suitable areas for *G. elata* varies substantially across future time periods. In comparison with the current distribution, the future distribution is expected to expand during the 2050s (to 75.18 × 10^4^ km^2^) and the 2090s (to 75.85 × 10^4^ km^2^), but contract during the 2070s (to 69.76 × 10^4^ km^2^), which account for 104.44%, 96.91%, and 105.37% of the current corresponding value, respectively. In the 2070s and 2090s, the expansion of highly suitable areas will be most pronounced in western Hubei and the Dabie Mountains of Anhui. In comparison with the distribution of *G. elata* in the 2050s and 2090s, the 2070s will see a significant contraction of highly suitable areas that will mainly occur at the border between Guizhou and northeastern Yunnan (Fig. 3).

### Shifts in the center of mass of highly suitable areas

**Figure 4:**
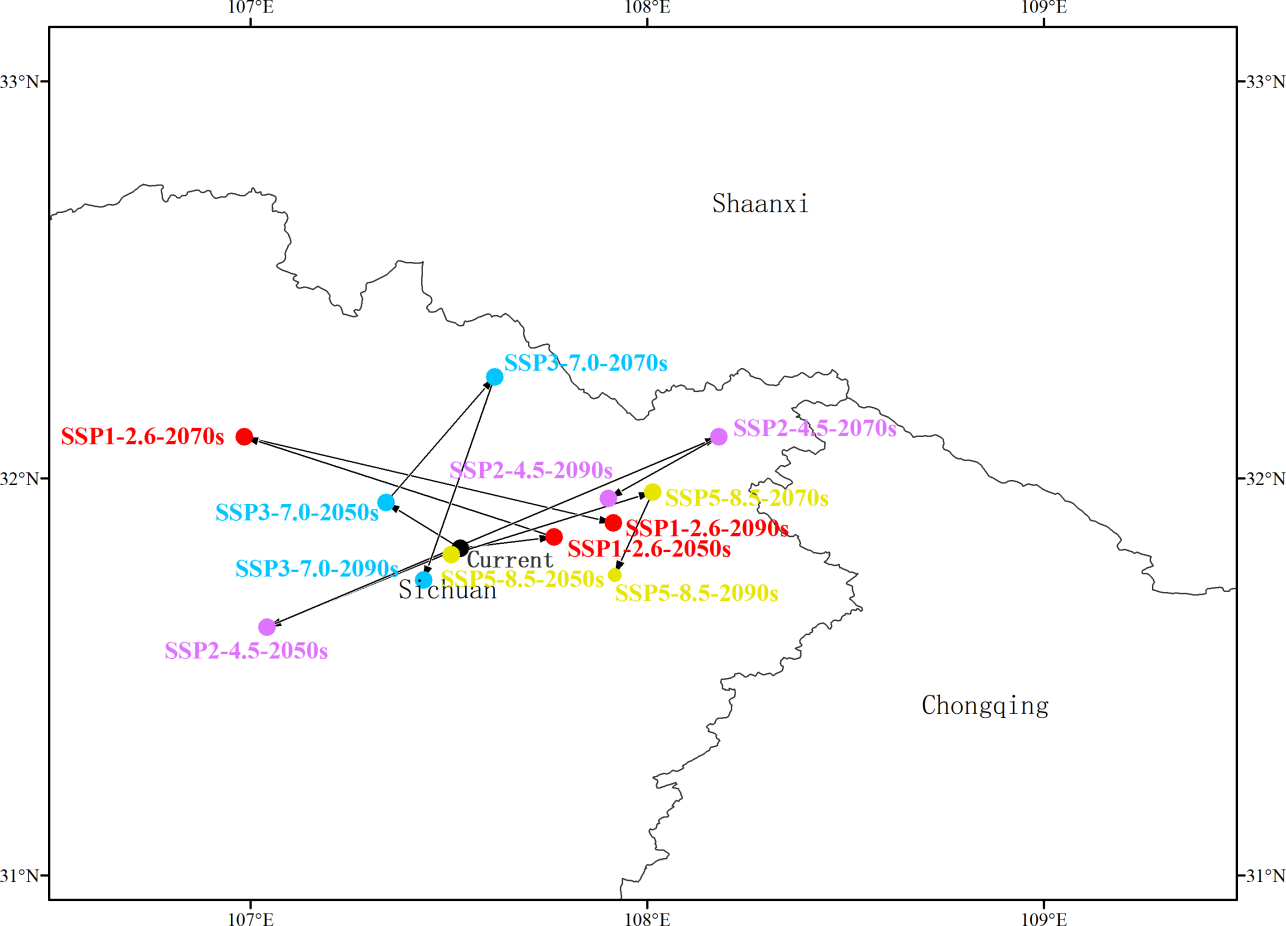
Variation in the centers of the distributions of highly suitable areas for *Gastrodia elata*. Arrows indicate the directions in which the centers of mass migrate in different time periods and for different climate change scenarios.

At present, the center of the distribution of highly suitable areas for *G. elata* is located at Pingchang, Bazhong, in the province of Sichuan (31°49′25.6008″N, 107°31′40.5228″E) (Fig. 4, Table S3). Under the SSP1−2.6 scenario, the center is expected to shift from Pingchang to Wanyuan (Dazhou, Sichuan) by the 2050s, then to Nanjiang (Bazhong, Sichuan) by the 2070s, and finally back to Wanyuan (Dazhou, Sichuan) in the 2090s. Similarly, under the SSP2−4.5 scenario, the center of the distribution of highly suitable areas in the 2050s will be located in Pingchang, while that during both the 2070s and the 2090s is expected to be in Wanyuan. Under the SSP3−7.0 scenario, the center of the distribution of highly suitable areas in both the 2050s and the 2070s is located in Tongjiang (Bazhong, Sichuan), and is expected to shift to Pingchang by the 2090s. Under the SSP5−8.5 scenario, the center of the distribution of highly suitable area is expected to move from Pingchang (2050s) to Wanyuan (2070s) and Xuanhan (2090s), respectively.

The above results indicate that the distribution center of highly suitable areas for *G. elata* is generally expected to move westward during the 2050s, except under the SSP1−2.6 scenario. The distance of this migration is minimized under the SSP5−8.5 scenario, where it corresponds to 2,764.89 m; while the greatest migration occurs under the SSP2−4.5 scenario, in which the distribution center is expected to move 51,728.81 m to the southwest from the current distribution center (Fig. 4, Table S3). In general, the distribution center of highly suitable areas for *G. elata* is expected to move northward in the 2070s and to the southwest in the 2090s.

### Change of climatic niche for *G. elata*

**Figure 5:**
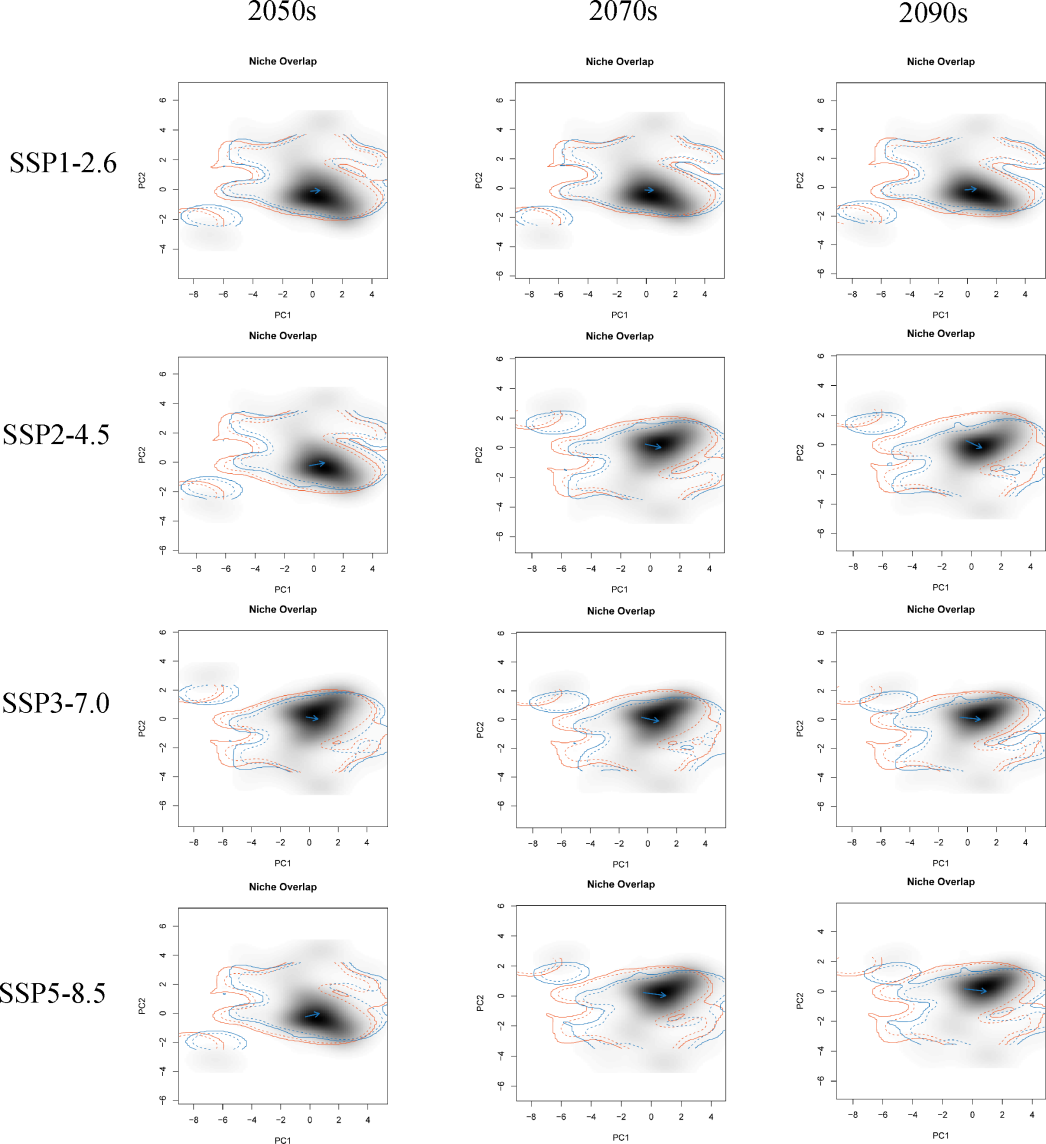
Predicted changes in the climatic niche of *Gastrodia elata* under different climate change scenarios. Orange lines indicate the climatic niche of *G. elata* in the current time period, while blue lines indicate its climatic niche in a future time period. Solid and dashed lines correspond to occupancy of 100% and 75% of the available environmental space, respectively. Darker shades indicate higher species densities. Blue arrows indicate how the center of the aspen climatic niche moves between the two ranges.

The first two principal components (PC1 and PC2) of all PCA analyses explained more than 60% of the selected parameter variables for the correlation analysis under different climate change scenarios (Fig. S4). These PCA analyses commonly showed that the future climatic niche center of *G. elata* will move towards the mean temperature of the coldest quarter, the minimum temperature of the coldest month, and the mean temperature of driest quarter (Fig. 5, Fig. S4). Moreover, the extent of the climatic niche changes of *G. elata* increase following time under different climatic scenarios, in which the minimal changes of the climatic niche present under the SPP1−2.6 scenario, while the largest occur under the SSP5−8.5 scenario (Fig. 5).

**Figure 6:**
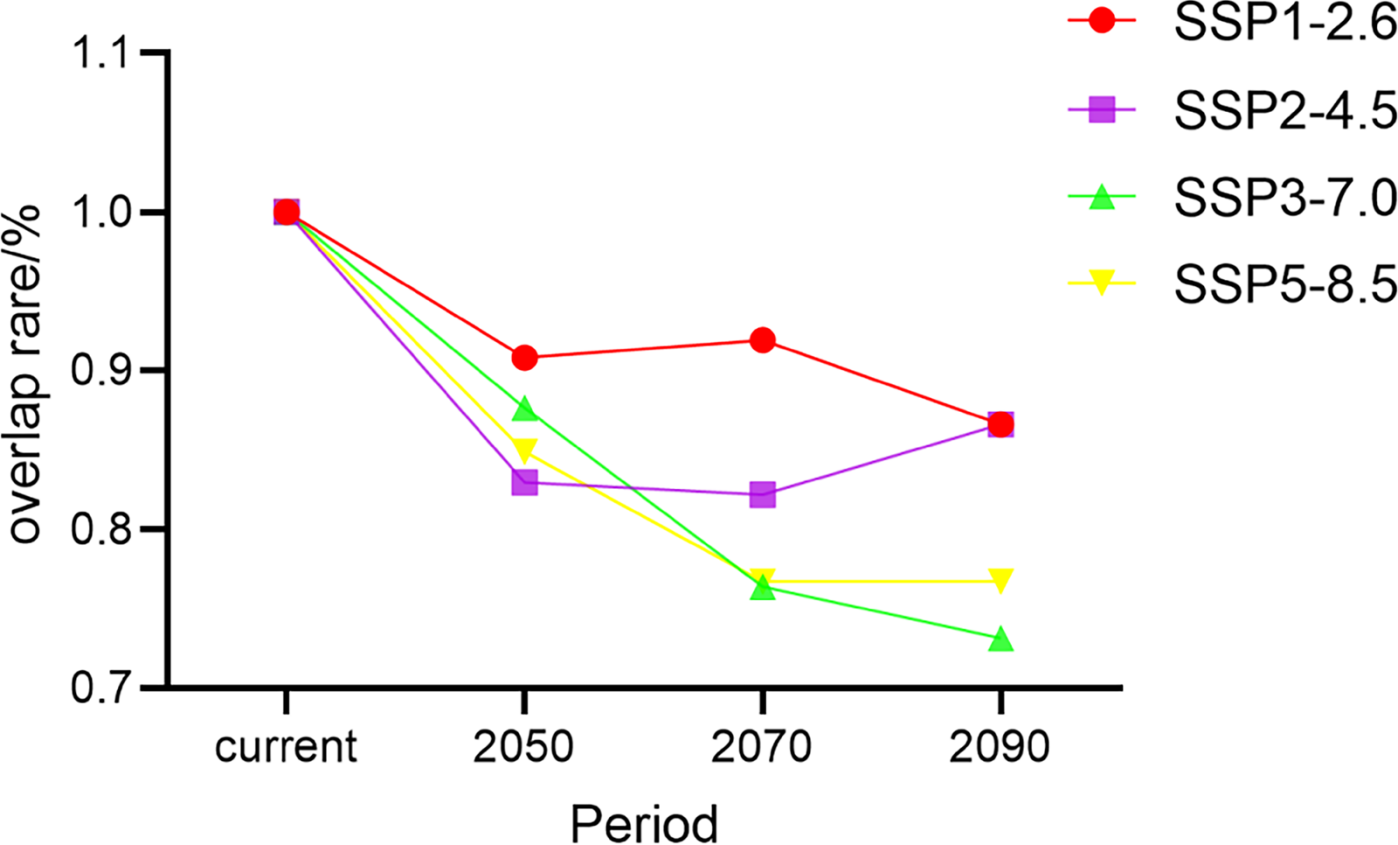
Changes in the rate of climatic niche overlap for *Gastrodia elata* in different time periods and under different climate change scenarios.

Schoener’s *D* is an index of a species’ ecological niches that are quantified based on its distributions in climate space (Quiroga, Premoli & Fernandez, 2018). In models for all four climate change scenarios, we found that Schoener’s *D* for *G. elata* in China tended to decrease over time. The rate of niche overlap rate decreased extensively under the SSP3−7.0 scenario and moderately under the SSP1−2.6 scenario (Fig. 6). Under the SSP2−4.5 scenario, Schoener’s *D* showed an increasing trend during the 2090s. The lowest niche overlap occurred between the current niches of *G. elata* and its niches under the SSP3−7.0 scenario in the 2090s (Schoener’s *D* = 0.73) (Table S4).

## Discussion

### Accuracy of species distribution models

Mapping the distributions of species and predicting how these will change in response to climate change are crucial for facilitating guidance on species conservatism and management (Hamid et al., 2018). In this study, we modelled the current distribution of suitable areas for *G. elata* in China, and predicted how this distribution would change in three future time periods under four different climate change scenarios. Our models displayed favorable accuracy and stability, with an average test AUC value of 0.947 for 10 repeat runs and the Kappa value of 0.817. Previous studies have reported that the Maxent method has the highest accuracy for assessing and predicting the effects of climate change on species distributions (Tang et al., 2021). However, the power of such model predictions is also known to be limited by several factors, such as the level of quality control exercised when determining sites of occurrence, the selection of bioclimatic factors, and the accuracy of model predictors (Zhao et al., 2021a; Zhao et al., 2021b). Aside from climatic variables, a range of other factors such as soil parameters, human activities, interspecific competition, and natural enemies can also impact the spatial distribution of species (Yang et al., 2021a; Yang et al., 2021b). The growth of *G. elata* cannot be separated from *Armillaria mellea*. As a kind of fungus, *A. mellea* is sensitive to temperature and humidity changes. In the modeling analysis, we could not consider the influence of *A. mellea* on the distribution of *G. elata.* In addition, Maxent models have mainly been based on the assumption that ecological niches are conserved over time, and therefore fail to consider the influence of evolutionary processes, which may consequently limit their accuracy and value (Aguirre-Liguori, Ramírez-Barahona & Gaut, 2021). Therefore, simulating models of species distributions that can comprehensively account for species’ ecological niches is key to the development of accurate and effective species distribution modelling.

### Effects of environmental factors on the distribution of *G. elata*

Environmental factors relating temperature and precipitation are often the most important parameters determining the growth and development of an organism, as well as the geographic distribution, diversity, and evolution of species (Remya, Ramachandran & Jayakumar, 2015; Zhao et al., 2021a; Zhao et al., 2021b). Our analysis using the Maxent model showed that the level of annual precipitation, altitude, and the mean temperature of the driest quarter were the primary factors shaping the distribution of *G. elata* in China (Table 1). In particular, we found that the level of annual precipitation and altitude contributed to over 50% of model predictions. Based on the response curve in our model, the optimal annual precipitation for the growth of *G. elata* ranges from 736.77–1,256.38 mm. This is consistent with studies on the biology of the species, which report that it is sensitive to humidity during its growth, and that soil moisture levels affects its growth and dormancy. *G. elata* is unlikely to go dormant during the winter if humidity levels are high. Hence, if the environmental conditions for dormancy are not met, the plant will display a weak growth of tuber buds following germination, or will rot after planting and fail to germinate altogether (Liu et al., 2017). Given this understanding of the biology of *G. elata*, it is unsurprising that no suitable areas for the species were found in places that experience higher levels of rain during the winter, such as in the mountainous area of southern Anhui and Wugong Mountain of Jiangxi.

We found that variations local temperature and precipitation as a result of changes in altitude generally affected the distribution of *G. elata* (Yan et al., 2022). The response curve for altitude indicated that the suitable altitudinal range for *G. elata* lay between 900 and 2,500 m (Fig. S3). This species would not grow normally in the plains of the middle and lower reaches of the Yangtze River, where the summer temperature exceeds the required temperature for its normal development (Liu et al., 2017). Similarly, we found that the Sichuan Basin did not contain suitable areas for *G. elata* owing to the relatively low altitudes of the region. *G. elata* begins to exhibit growth at temperatures of 10–12 °C, and grows rapidly at 20–25 °C. The growth of *G. elata* is inhibited when environmental temperatures continuously exceed 30 °C, such as during the summer. The tubers of *G. elata* only germinate in the second year after the plant experiences a period of dormancy at low temperatures (within the range of 0–5 °C) during the previous winter.

### Effects of climate change on the distribution of *G. elata*

In response to climate change, plant species can continue to survive and grow through physiological adaptations, or otherwise shift their distributions to more climatically suitable areas (Kong et al., 2021; Xu et al., 2019). One key aspect of ongoing climate change is the widespread effect of climate warming in driving the migration of species northward (Boisvert-Marsh & de Blois, 2021). In this study, we used Maxent and ArcGIS to map the current distribution and predict the future distribution of *G. elata* in China. Our forecasts show that the distribution of suitable areas for *G. elata* its likely to move northward over time—a trend that is consistent with the responses of many other plant species experiencing global warming (Fig. 4). For example, under the SSP1−2.6 scenario, the distribution of highly suitable areas for *G. elata* is expected to shift northwards. Under the SSP2−4.5 scenario, the distribution of highly suitable areas for *G. elata* in Yunnan, Sichuan, Guizhou, and Hubei is likely to contract, while that in northern Shaanxi is expected to expand (Fig. 3).

Using three ecological niche models (BIOCLIM, DOMAIN and MAXENT), Zhang et al. (2017) found that most suitable areas for *G. elata* during the current time period were mainly distributed around the Sichuan basin and the central-eastern regions in China. In contrast, the results of our study suggest that highly suitable areas for *G. elata* are mainly distributed in central Sichuan, along the border between Shaanxi and Sichuan, as well as in western Hubei, northeastern Yunnan, and in the Chongqing Municipality (Fig. 3). These areas experience temperate monsoon climates, which are characterized by hot summers with high rainfall, while cold and dry winters. Although *G. elata* is a widely temperate species, it cannot tolerate high temperatures and extremely cold weather. Therefore, the species cannot grow in locations where summer temperatures remain consistently hot, as well as those where winter temperatures are insufficiently cold. This explains the lack of suitable areas for *G. elata* in southern Yunnan and most parts of Guangdong. Our results further show that under future climate change areas that are low suitable for *G. elata* are likely to expand in regions such as Liaoning, Jilin, and Shanxi. This may be related to the future northward shift of the main precipitation belt with increasing annual precipitation in northern and western China in the future (Yang et al., 2021a; Yang et al., 2021b).

### Effects of climate change on niche of *G. elata*

The rapid development of statistical environmental models and geoinformation technology has greatly advanced efforts to understand the impacts of climate change on the ecological niches of species. One goal of our study was to compare the current and future ecological niches of *G. elata*, and examine if any shifts in the species’ ecological niche were likely to occur over time. The environmental variable-related PCA analysis revealed that the ecological niche space of *G. elata* varied moderately changes over time and under different climate change scenarios. We found that the environmental variables during changes in *G. elata*’s ecological niche were primarily related to the minimum temperature of the environment. When species are unable to adapt to new environmental conditions, species can disperse to new areas, adapt *in situ*, or become locally extinct (Villaverde et al., 2017; Christmas, Breed & Lowe, 2016). A value of Schoener’s *D* that is below 95% indicates a change in a species’ ecological niche (Zhu et al., 2021). Our models revealed the *Schoener’s D* values ranging from 73% to 90% between all two comparisons of the current and future distributions of *G. elata*. This indicates that the current potential suitable areas for *G. elata* would be reduced in the future. These results on the ecological niche dynamics of *G. elata* are consistent with the results from our SDMs, and indicate that areas that are currently highly suitable for *G. elata* are likely to contract in the future. Our results also suggests that *G. elata* may shift its distribution to areas at higher latitudes or higher altitudes in response to increased climate warming.

### Conservation of wild resources

*G. elata* has been used as a medicinal resource in China for more than 2,000 years (Ahn et al., 2007). As a result of overexploitation and area degradation, wild populations of *G. elata* in China have decreased continuously, and the species is currently classified as “vulnerable” by the International Union for Conservation of Nature. Because of its high medicinal value, there is a high demand for *G. elata* in the commercial market (Ahn et al., 2007). To meet increasing demands for *G. elata* products, artificial cultivation of the species was realized in the 1970s. To grow *G. elata*, populations of *A. mellea* are first introduced to break down woody material within the area and then infected *G. elata* to provide nutrients. Large-scale cultivation of *G. elata* leads to massive deforestation and area degradation, and causes the destruction of wild populations (Chen et al., 2014).

To mitigate the environmental damages caused by the massive cultivation of *G. elata*, we should explore more sustainable models of cultivation, which incorporate a better understanding of the potential distribution of the species. Our model predictions showed that the distribution of highly suitable areas for *G. elata* are likely to remain relatively stable under different climate change scenarios in several areas such as southern Shaanxi, western Hubei, northeastern Yunnan, central Sichuan, Chongqing, and Guizhou. That is, the growth of *G. elata* in these areas are less likely to be affected by future climate warming. Hence, it is imperative to strengthen the *in situ* conservation of wild *G. elata* populations in these areas and mitigate any impacts of human disturbance. During the whole growth process, *G. elata* does not need to undergo photosynthesis to provide nutrients; hence, it can be grown indoors in low suitable or even unsuitable conditions to create suitable climate for the growth of *G. elata*. Thus, conserving wild germplasms of this species and establishing key germplasm repositories will be critical for of the effective conservation and sustainable use of *G. elata* in the future.

## Conclusions

In this study, we used the Maxent model to identify the distribution of potentially suitable areas for *G. elata* in China at present, in the future, and under a variety of climate change scenarios. The results show that the level of annual precipitation and altitude are the most important factors influencing the distribution of *G. elata*. Under different climate change scenarios, areas that are highly suitable and relatively stable for the growth of *G. elata* are mainly found in Shaanxi, Hubei, Sichuan and Yunnan. We also found that the center of *G. elata* ecological niche is likely to shift toward the mean temperature of the coldest quarter, the minimum temperature of the coldest month, and the mean temperature of the driest quarter. Over time, the overlap in the ecological niche of *G. elata* is expected to decrease, and most extensively under the SSP 7.0 scenario. Overall, our study provides important empirical knowledge to guide the conservation and sustainable cultivation of *G. elata* resources in the future.

##  Supplemental Information

10.7717/peerj.15741/supp-1 Supplemental Information 1 Reliable test of the distribution model created for *Gastrodia elata*.

10.7717/peerj.15741/supp-2 Supplemental Information 2 Jackknife test for evaluating the relative importance of environmental variables for *Gastrodia elata*

10.7717/peerj.15741/supp-3 Supplemental Information 3 Response curves showing the important environmental factors affecting the distribution of *Gastrodia elata*.

10.7717/peerj.15741/supp-4 Supplemental Information 4 Correlation and principal component contribution of each factor involved in PCA-env analysis

10.7717/peerj.15741/supp-5 Supplemental Information 5 Data points information on the distribution of *Gastrodia elata*

10.7717/peerj.15741/supp-6 Supplemental Information 6 Changes of habitats of high suitability for *Gastrodia elata* in different periods

10.7717/peerj.15741/supp-7 Supplemental Information 7 The centroid of highly suitable regions under different climate scenario

10.7717/peerj.15741/supp-8 Supplemental Information 8 Niche comparisons and variation in principle components PC1 and PC2 between current and future projected distribution range of *G. elata*.

## Additional Information and Declarations

### Data Availability

The following information was supplied regarding data availability: The raw measurements are available in the Supplemental Files. Third party data was available at the following: CVH (http://www.cvh.ac.cn); GBIF (http://www.gbif.org); SRSPE (http://mnh.scu.edu.cn); PPBC (http://ppbc.iplant.cn); WorldClim (http://www.worldclim.org/).
