# Supplementary figures and images for: Impact of climate change on the geographical distribution and niche dynamics of Gastrodia elata
Source: PeerJ. 2023 Jul 24;11:e15741. doi: 10.7717/peerj.15741 (PMC10373646; doi:10.7717/peerj.15741)

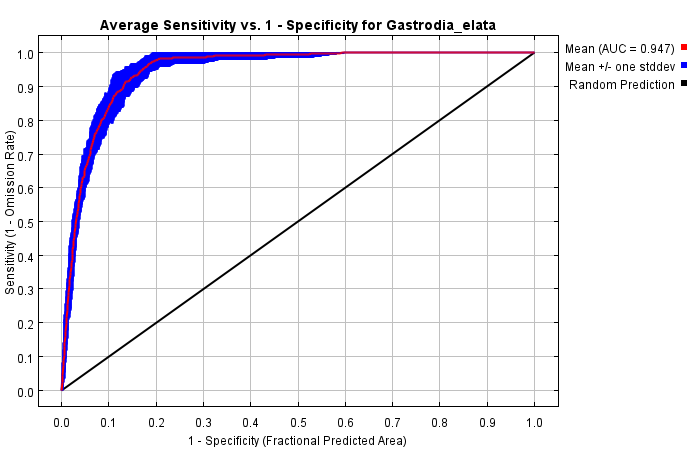

Supplement: Supplemental Information 1 [file peerj-11-15741-s001.png]

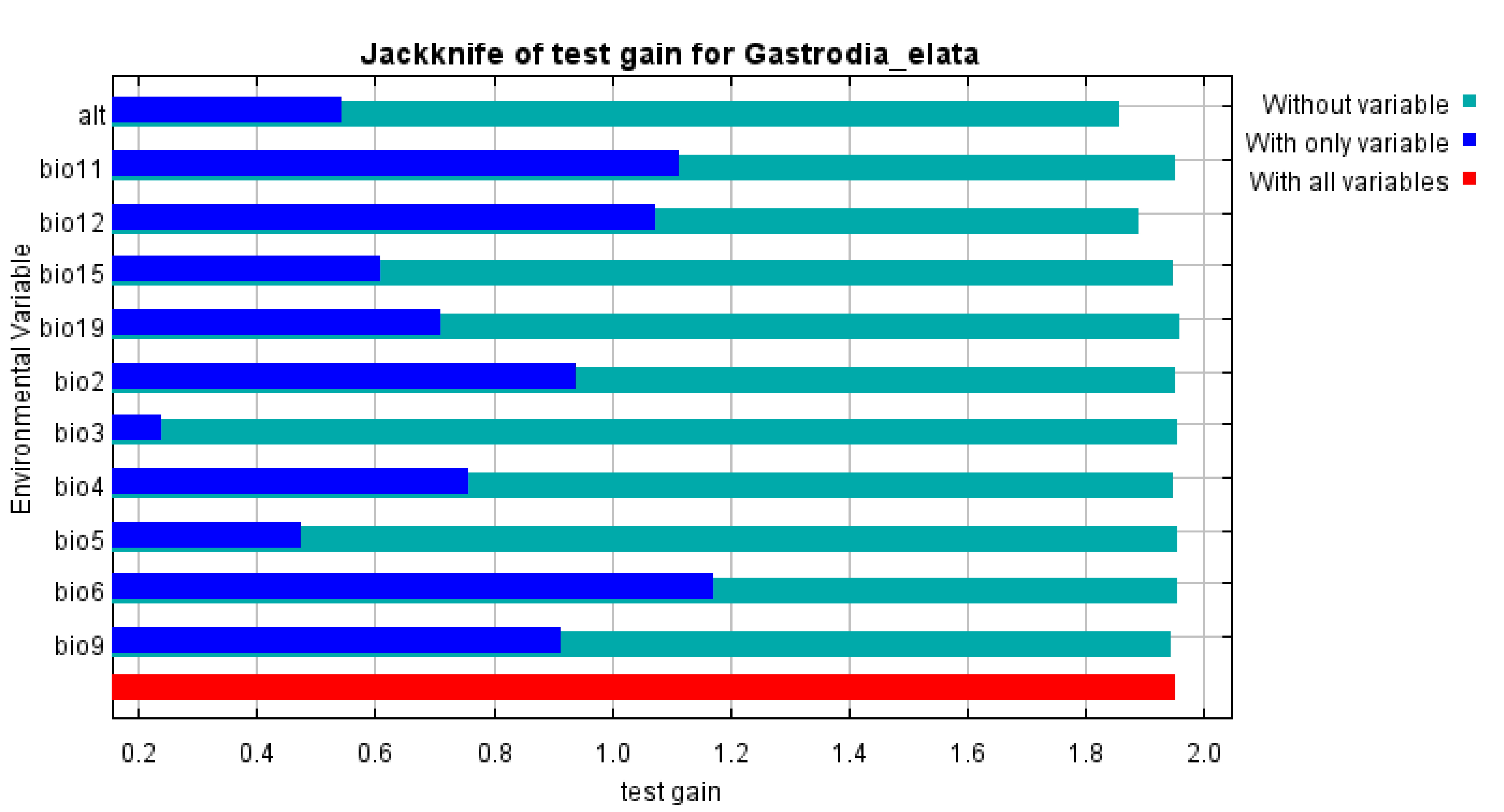

Supplement: Supplemental Information 2 [file peerj-11-15741-s002.png]

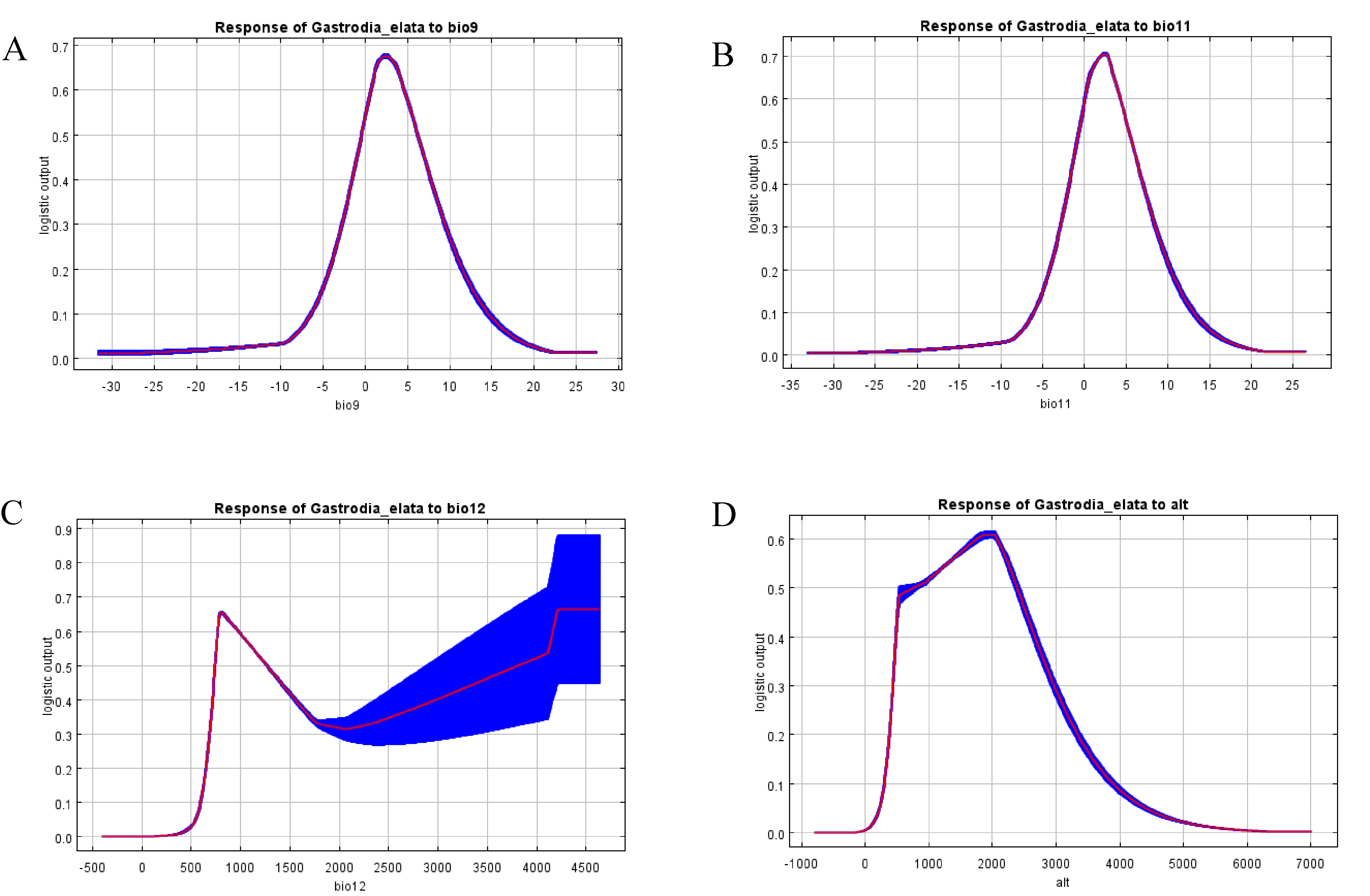

Supplement: Supplemental Information 3 [file peerj-11-15741-s003.png]

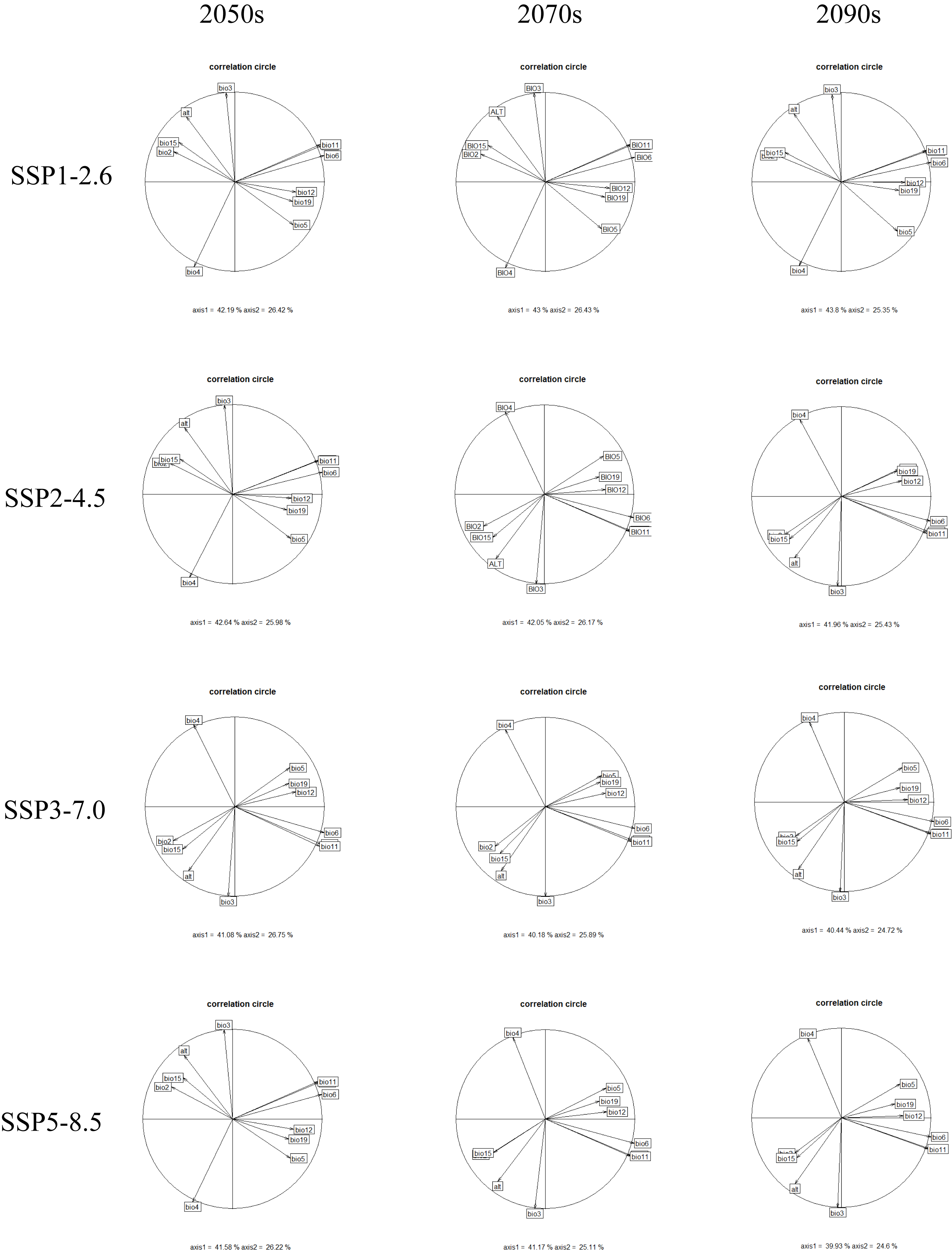

Supplement: Supplemental Information 4 [file peerj-11-15741-s004.png]
