# Supplementary material for: Impact of climate change on the geographical distribution and niche dynamics of Gastrodia elata
Source: PeerJ. 2023 Jul 24;11:e15741. doi: 10.7717/peerj.15741 (PMC10373646; doi:10.7717/peerj.15741)
Supplement: Supplemental Information 6 [file peerj-11-15741-s006.docx]

**Table S2:** Changes of habitats of high suitability for *G. elata* in the different periods.

| Area (km^2^) | | Expansion | No Change | Contraction |
| --- | --- | --- | --- | --- |
| SSP1-2.6 | current-2050s | 2454.51 | 65964.91 | 10620.47 |
|  | current-2070s | 1250.88 | 45691.61 | 30893.76 |
|  | current-2090s | 1958.89 | 53645.16 | 22940.21 |
| SSP2-4.5 | current-2050s | 2147.69 | 50081.41 | 26503.97 |
|  | current-2070s | 8071.55 | 58483.37 | 18101.99 |
|  | current-2090s | 2147.69 | 57444.93 | 19140.44 |
| SSP3-7.0 | current-2050s | 826.04 | 60277.05 | 16308.32 |
|  | current-2070s | 2619.72 | 48122.52 | 28462.85 |
|  | current-2090s | 3445.75 | 63581.20 | 13004.17 |
| SSP5-8.5 | current-2050s | 2289.30 | 58601.38 | 17983.99 |
|  | current-2070s | 2619.72 | 48122.52 | 28462.85 |
|  | current-2090s | 5215.83 | 61527.91 | 15057.46 |
