# Supplementary material for: Impact of climate change on the geographical distribution and niche dynamics of Gastrodia elata
Source: PeerJ. 2023 Jul 24;11:e15741. doi: 10.7717/peerj.15741 (PMC10373646; doi:10.7717/peerj.15741)
Supplement: Supplemental Information 7 [file peerj-11-15741-s007.docx]

**Table S3:** The centroid of highly suitable regions under different periods.

| Period | | Longitude | Latitude | Distance (m) |
| --- | --- | --- | --- | --- |
| current | | 107.527923 | 31.823778 | 0 |
| SSP1-2.6 | 2050s | 107.764293 | 31.853112 | 23028.67 |
|  | 2070s | 106.983324 | 32.104682 | 80154.43 |
|  | 2090s | 107.914011 | 31.888003 | 92830.49 |
| SSP2-4.5 | 2050s | 107.04064 | 31.625713 | 51728.81 |
|  | 2070s | 108.180268 | 32.104682 | 121672.20 |
|  | 2090s | 107.901297 | 31.950147 | 31717.38 |
| SSP3-7.0 | 2050s | 107.340927 | 31.939808 | 22015.93 |
|  | 2070s | 107.614488 | 32.256283 | 43305.52 |
|  | 2090s | 107.435316 | 31.744366 | 58217.92 |
| SSP5-8.5 | 2050s | 107.505586 | 31.80786 | 2764.89 |
|  | 2070s | 108.013833 | 31.966118 | 51970.04 |
|  | 2090s | 107.917051 | 31.757406 | 24544.55 |
