# Supplementary material for: Impact of climate change on the geographical distribution and niche dynamics of Gastrodia elata
Source: PeerJ. 2023 Jul 24;11:e15741. doi: 10.7717/peerj.15741 (PMC10373646; doi:10.7717/peerj.15741)
Supplement: Supplemental Information 8 [file peerj-11-15741-s008.docx]

**Table S4:** Niche comparisons and variation in principle components PC1 and PC2 between current and future projected distribution range of *G. elata*.

| Niche comparison pairs | | Niche overlap (D) | PC1 (%) | PC2 (%) |
| --- | --- | --- | --- | --- |
| SSP1-2.6 | current-2050s | 0.90 | 42.19 | 26.42 |
|  | current-2070s | 0.91 | 43.00 | 26.43 |
|  | current-2090s | 0.86 | 43.80 | 25.35 |
| SSP2-4.5 | current-2050s | 0.83 | 42.64 | 25.98 |
|  | current-2070s | 0.82 | 42.05 | 26.17 |
|  | current-2090s | 0.75 | 41.96 | 25.43 |
| SSP3-7.0 | current-2050s | 0.87 | 41.08 | 26.75 |
|  | current-2070s | 0.76 | 40.18 | 25.89 |
|  | current-2090s | 0.73 | 40.44 | 24.72 |
| SSP5-8.5 | current-2050s | 0.84 | 41.58 | 26.22 |
|  | current-2070s | 0.76 | 41.17 | 25.11 |
|  | current-2090s | 0.74 | 39.93 | 24.60 |
